# Functional *5Bq* allele is responsible for the compact spike phenotype in common wheat (*Triticum aestivum* L.)

**DOI:** 10.1186/s12870-026-08583-x

**Published:** 2026-03-17

**Authors:** Qingcheng Li, Yang Li, Jing Zhu, Zhenru Guo, Yuqing Che, Xin Chen, Linlin Zhou, Shenglong Chen, Ting Gao, Li Kong, Yunfeng Jiang, Guoyue Chen, Qiantao Jiang, Yazhou Zhang, Qiang Xu, Yuming Wei, Jian Ma, Aili Li, Long Mao, Youliang Zheng, Qing Chen, Pengfei Qi

**Affiliations:** 1 State Key Laboratory of Crop Gene Exploration and Utilization in Southwest China, Sichuan Agricultural University, Chengdu, 611130 China; 2 Triticeae Research Institute, Sichuan Agricultural University, Chengdu, 611130 China; 3 Institute of Crop Science, Chinese Academy of Agricultural Sciences, Beijing, 100081 China

**Keywords:** Compact spike, *Q* gene, MicroRNA172, Mutation, Breeding

## Abstract

**Background:**

Spike morphology is an important characteristic of common wheat because of its effects on yield potential. However, few available genes regulating spike morphology have been reported in common wheat.

**Results:**

We identified a compact spike mutant *S-Cp2*, which was obtained by treating common wheat cultivar ‘Shumai482’ with ethyl methanesulfonate. The gene responsible for the compact spike phenotype of *S-Cp2* was mapped and cloned. According to a genetic analysis, the formation of a compact spike in *S-Cp2* was controlled by a single dominant locus (*Cs-5B*) on chromosome 5BL. This locus was mapped to an 87 kb interval between markers *KASP-58070* and *KASP-58158*, wherein *5Bq* gene was detected. One missense mutation was called within the microRNA172-binding site of *5Bq* in *S-Cp2*, suggesting that the mutated *5Bq* allele (*5BQ*^*4*^) is responsible for the compact spike phenotype. This was confirmed by cloning *5Bq* in revertants obtained by treating *S-Cp2* with ethyl methanesulfonate. As expected, the *5BQ*^*4*^ allele was expressed at significantly higher levels than the *5Bq* allele in developing spike tissues. According to a haplotype analysis, the functional haplotype 1 for *5Bq* was gradually accumulated in modern Chinese wheat cultivars.

**Conclusion:**

In this study, we identified a functional *5Bq* allele by genetic mapping, which was responsible for the compact spike phenotype in mutant *S-Cp2*. The study findings highlight the potential utility of *5Bq* for breeding novel wheat cultivars.

**Supplementary Information:**

The online version contains supplementary material available at 10.1186/s12870-026-08583-x.

## Introduction

Common wheat (*Triticum aestivum* L.) is one of the most important food crops worldwide. Common wheat provides approximately 20% of the calories and 22% of the proteins consumed by humans and feeds more than 35% of the global population [1–3]. Wheat is largely consumed by humans after being processed into bread and other foods. The demand for wheat will continue to grow as the cultivated land area decreases and the global population increases [4–6]. Therefore, one of the primary goals of wheat breeders and growers is increasing the grain yield, which will affect the profitability of wheat production as well as food security [7–10].

The spike is an important wheat plant part because it is where grains develop and mature. Spike morphological traits are closely associated with wheat grain yield potential and stability, making them important agronomic traits for common wheat domestication and breeding [11–15]. Spike number per unit area, grain number per spike, and thousand kernel weight (TKW) are important yield components in common wheat [16–21]. Increases in grain number per spike and TKW generally lead to an increase in grain yield, which is also correlated with spike morphological traits, including rachis internode length, rachis length, and glume length [22–25].

Spike morphology has attracted the interest of researchers [11, 26–30]. Several spike-related genes have been characterized, including the well-known genes *Q*, *C*, and *S*. The *Q* gene, located on chromosome 5AL, is involved in regulating rachis length, rachis internode length, and grain number per spike [31, 32]. The *C* gene, located on chromosome 2DS, has pleiotropic effects on rachis internode length and grain shape [27, 30]. The *S* gene on chromosome 3DS defines grain shape as well as spikelet and rachis length in wheat [33, 34]. In addition to influencing spike morphology, *Q*, *C*, and *S* also have pleiotropic effects on yield-related traits.

In common wheat, *Q*, one of the most important domestication genes, encodes an APETALA2-like (AP2-like) transcription factor [31, 32]. The domesticated *Q* allele arose from a spontaneous mutation in the microRNA172-binding region of the *q* allele during wheat evolution. *Q* influences many domestication-related traits, including threshability, plant height, rachis fragility, and flowering time [35–40]. Moreover, *Q* is an ideal target for wheat breeders because its favorable alleles can increase the grain protein content, TKW, and grain yield [3, 41, 42]. *Q* has homoeologs on wheat chromosomes 5BL (*5Bq*) and 5DL (*5Dq*). Similar to the *Q* gene on 5AL [43, 44], an introduced point mutation in the microRNA172-binding region of *5Dq* resulted in pleiotropic effects on rachis internode length and plant dwarfism [45]. Another study revealed that *5Bq* is a pseudogene because it does not encode a full-length functional protein [46]. A functional *5Bq* allele has yet to be reported.

In this study, we report a compact spike mutant, *S-Cp2*, screened from an ethyl methanesulfonate (EMS)-treated population of common wheat cultivar ‘Shumai482’. The spike morphology of the *S-Cp2* mutant was similar to that of club wheat [27]. The objective of the present study is to identify the locus (named *Cs-5B*)/gene (*5Bq*) responsible for the compact spike phenotype (assessed by rachis internode length) in *S-Cp2*. This research deepens our understanding of the formation of wheat spike morphology, and provides new knowledge for wheat breeding.

## Materials and methods

### Plant materials and growth conditions

The *S-Cp2* mutant with a compact spike was isolated from a common wheat cultivar ‘Shumai482’ M_2_ population (i.e., second generation after a treatment with 0.6% EMS; Sigma-Aldrich, St. Louis, MO, USA). The *S-Cp2* mutant was crossed six times with ‘Shumai482’, after which homozygous *S-Cp2* was compared with wild-type (WT; ‘Shumai482’) plants to examine mutant properties (Supplementary Fig. S1). *S-Cp2* seeds were treated with 0.4% EMS to obtain revertants.

‘QZ196’ is a common wheat line with significant differences in spike morphology and genetic background from *S-Cp2*. Therefore, *S-Cp2* was used as the male parent for a cross with ‘QZ196’ to construct a segregating population. The F_2_ population derived from the ‘QZ196’ × *S-Cp2* cross was used for mapping. The F_3_ population comprising 2,450 individuals derived from the F_2_ heterozygous individuals (‘QZ196’ × *S-Cp2*) was used for fine-mapping. Another two F_2_ populations were prepared by crossing *S-Cp2* (male) with the common wheat cultivars ‘Mianmai112’ and ‘Shumai133’ (Supplementary Fig. S1). Wheat plants were grown at the experimental farm of Sichuan Agricultural University in Wenjiang (103° 51ʹ E, 30° 43ʹ N), with a row spacing of 20 cm × 10 cm. *S-Cp2* and WT plants were grown in a 2 m × 2 m plot with a row spacing of 20 cm × 5 cm during the 2022–2023 and 2023–2024 wheat growing seasons. A nitrogen: phosphorous: potassium (15:15:15) compound fertilizer was applied (750 kg/ha) before sowing. Local pest control and weed management practices were applied. Rachis internode length was determined by calculating the ratio of the rachis length to the rachis node number at GS87 (a decimal code system for wheat development) [47], as described by Nanape [48]. In this study, all plants were visually distinguished on the basis of two distinct spike morphotypes (compact spike and normal spike). Compact and normal spikes were defined as those with a rachis internode length ≤ 3.20 mm and > 4.80 mm, respectively; there were no spikes with a rachis internode length between 3.20 mm and 4.80 mm in our populations (Supplementary Fig. S2). The non-overlapping bimodal distribution shows that the compact and normal spikes in our populations can be clearly differentiated.

A total of 696 common wheat materials, including 329 Chinese landraces (CL), 99 modern Chinese cultivars released before the year of 2000 (MCCB), and 268 modern Chinese cultivars released after 2000 (MCCA; Supplementary Table S1), were used for the haplotype analysis. Twenty seeds for each material were germinated in Petri dishes, after which leaves were collected from seedlings at GS10 for the subsequent extraction of DNA. In addition, partial common wheat materials, including 122 CL, 92 MCCB, and 90 MCCA, were grown at the experimental farm of Chinese Academy of Agricultural Sciences in Xinxiang (113° 54ʹ E, 35° 18ʹ N) during the 2023–2024 wheat growing seasons for the measurement of rachis internode length. Each common wheat was grown in a 2.5 m × 2 m area, with a row spacing of 25 cm × 10 cm.

### Examination of agronomic traits

*S-Cp2* and WT plants were randomly selected at GS87 for an analysis of the following agronomic traits: plant height (cm), rachis length (cm), rachis node number, grain number per spike, flag leaf length (cm), flag leaf width (cm), and effective tiller number. Rachis internode length (mm) was calculated as the ratio of rachis length (mm) to rachis node number. An SC-G automatic seed analyzer (Wanshen, Hangzhou, China) was used to measure the post-harvest TKW (g), grain length (mm), and grain width (mm).

### Mapping

Genomic DNA was extracted from fresh leaves according to the cetyltrimethylammonium bromide method [49]. Equal amounts of genomic DNA isolated from 30 homozygous individuals with a compact spike and 30 plants with a normal spike in the ‘QZ196’ × *S-Cp2* F_2_ population were used to produce two pooled genomic DNA samples for the bulked segregant analysis coupled with exome capture sequencing (BSE-seq), which was performed using a wheat exome capture panel (Tcuni Bioscience, Chengdu, China) that contained a 268.9 Mb capture space and covered 107,400 high-confidence genes. DNA samples were sequenced using the Illumina HiSeq Nova platform (150-bp paired-end reads). The paired reads were mapped to the Chinese Spring v1.0 reference sequence [50]. Candidate genomic regions associated with compact spike phenotype were identified on the basis of single nucleotide polymorphism (SNP) density and Euclidean distance (ED) [51, 52]. These analyses were performed using the web-based BSA platform WheatGmap (https://www.wheatgmap.org) [53, 54]. SNPs in the two pools were converted into kompetitive allele-specific PCR (KASP) markers, which were used to genotype the mapping population (http://www.polymarker.info) [55]. PCR amplifications were completed in a 10 µL reaction volume using HiGeno 2 × Probe Mix B (JasonGen, Beijing, China). All experiments were performed according to the manufacturer’s instructions. Selected markers along with JoinMap (version 4.0) were used to generate a genetic map [56].

### Gene cloning and expression analysis

Total RNA was extracted and then reverse transcribed to cDNA using a Plant RNA Extraction kit (version 1.5) (Biofit, Chengdu, China) and a HiScript III 1st Strand cDNA Synthesis Kit (Vazyme, Nanjing, China), respectively. Both kits were used as recommended by the manufacturers.

Primers (Supplementary Table S2) were designed according to ‘Chinese Spring’ and ‘Kenong 9204’ reference sequences [50, 57] using Premier 5.0 (Premier Biosoft, San Francisco, CA, USA). PCR amplifications were performed in a 50 µL reaction volume using Phanta Mix Super-Fidelity DNA Polymerase P505 (Vazyme) and a Veriti™ 96-Well Thermal Cycler. The PCR conditions were as follows: 95 °C for 5 min; 35 cycles of 95 °C for 30 s, 60 °C for 30 s, and 72 °C for 90 s; 72 °C for 10 min; and 12 °C (hold). Amplified target fragments were inserted into the pCE2 TA/Blunt-Zero vector using the 5 min TA/Blunt-Zero Cloning Kit (Vazyme). Positive colonies were sequenced by Sangon Biotech (Chengdu, China). The cloning and sequencing experiments were repeated at least three times. Sequences were analyzed using DNAMAN (version 8) (Lynnon Biosoft, San Ramon, CA, USA).

*S-Cp2* and WT plants were grown in a glasshouse with a 12-h light (25 °C):12-h dark (20 °C) cycle. Root, stem, and leaf samples were collected at GS20. Developing spikes were collected at GS22, GS24, and GS29. Developing grains were collected at 5, 10, and 15 days post-anthesis (DPA). *5Bq* expression levels in *S-Cp2* and WT plants were analyzed by qRT-PCR, which was completed using the Hieff^®^ qPCR SYBR Green Master Mix (Yeasen Biotechnology, Shanghai, China), with wheat *GAPDH* (GenBank No. XM_044566753) and *β-tubulin* (GenBank No. NM_001427965) genes selected as the reference controls for normalizing expression data (Supplementary Table S2). All experiments were completed according to the manufacturer’s instructions.

### Subcellular localization

The coding sequences of the *5Bq*, *5BQ*^*4*^, and *5BQ*^*4*^*-N9* alleles lacking termination codon were fused with the pCAMBIA2300-eGFP (enhanced green fluorescent protein, eGFP) vector. Primer details are provided in Table S2. The resulting pCAMBIA2300-5Bq-eGFP, pCAMBIA2300-5BQ^4^-eGFP, and pCAMBIA2300-5BQ^4^-N9-eGFP vectors were transformed into *Agrobacterium tumefaciens* strain GV3101. The transformed strains with the corresponding constructs were infiltrated into *Nicotiana benthamiana* leaves via *A. tumefaciens-*mediated method [58]. After 24 h of infiltration, the fluorescent signals were observed by STELLARIS STED/EM CPD300 confocal microscope (Leica, Wetzlar, Germany).

### Data analysis

Data were calculated and compiled using Excel 2010 (Microsoft 2010, Redmond, WA, USA). IBM SPSS Statistics 26 (IBM Corporation, Armonk, NY, USA) was used for Student’s *t*-test, Chi-squared (χ^2^) test, analysis of variance (ANOVA), and Pearson’s correlation analyses. Data were recorded as the mean ± standard deviation. Plots were produced using Origin 2024 (OriginLab., Northampton, MA, USA) and R version 4.4.1 with the ggplot2 package.

## Results

### The compact spike phenotype of *S-Cp2* was controlled by a single dominant nuclear locus

**Fig. 1:**
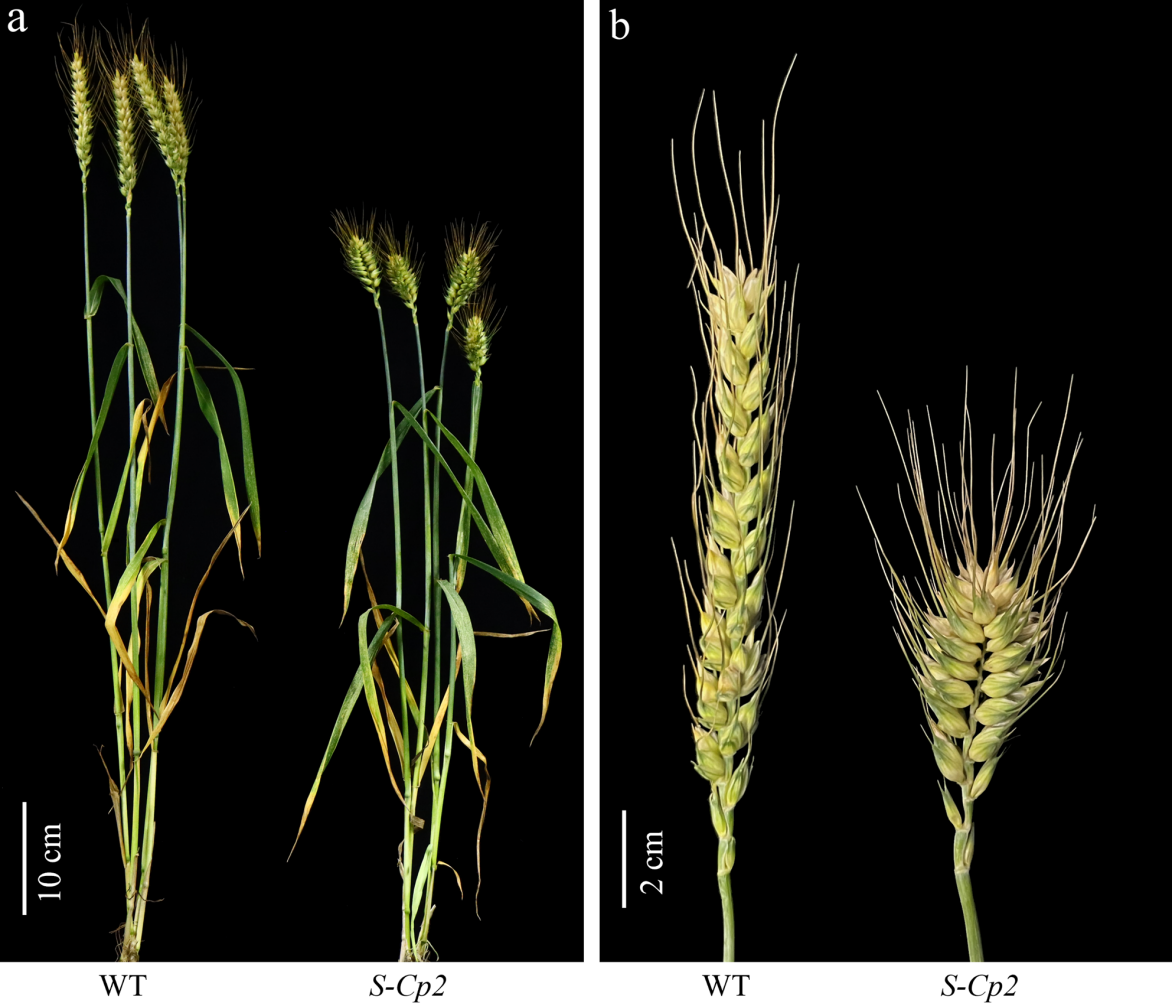
Comparison of the agronomic traits of *S-Cp2* and WT plants. **a** *S-Cp2* and WT plants at GS80. Scale bar, 10 cm. **b** Spikes of *S-Cp2* and WT plants at GS80. Scale bar, 2 cm

*S-Cp2* had a compact spike similar to that of club wheat (Fig. 1; Supplementary Table S3). The *S-Cp2* mutant was reciprocally backcrossed with ‘Shumai482’ to clarify the genetic basis of the compact spike phenotype. All BC_1_F_1_ plants had a compact spike, suggesting that the mutant phenotype was controlled by at least one nuclear gene. *S-Cp2* was crossed with three common wheat lines (i.e., ‘QZ196’, ‘Mianmai112’, and ‘Shumai133’) with a normal spike. In the ‘QZ196’ × *S-Cp2* F_2_ population, 134 plants had a compact spike and 46 plants had a normal spike, which was consistent with the theoretical 3:1 segregation ratio (χ^2^ = 0.03 < χ^2^_0.05_ = 3.84). Similarly, the corresponding ratios in the ‘Mianmai112’ × *S-Cp2* F_2_ population (207 plants with a compact spike and 73 plants with a normal spike; χ^2^ = 0.17 < 3.84) and the ‘Shumai133’ × *S-Cp2* F_2_ population (174 plants with a compact spike and 55 plants with a normal spike; χ^2^ = 0.12 < 3.84) also fit the expected 3:1 segregation ratio. These results suggest that the compact spike phenotype of *S-Cp2* was due to a single dominant nuclear mutation. 

### Mapping of *Cs-5B*

**Fig. 2:**
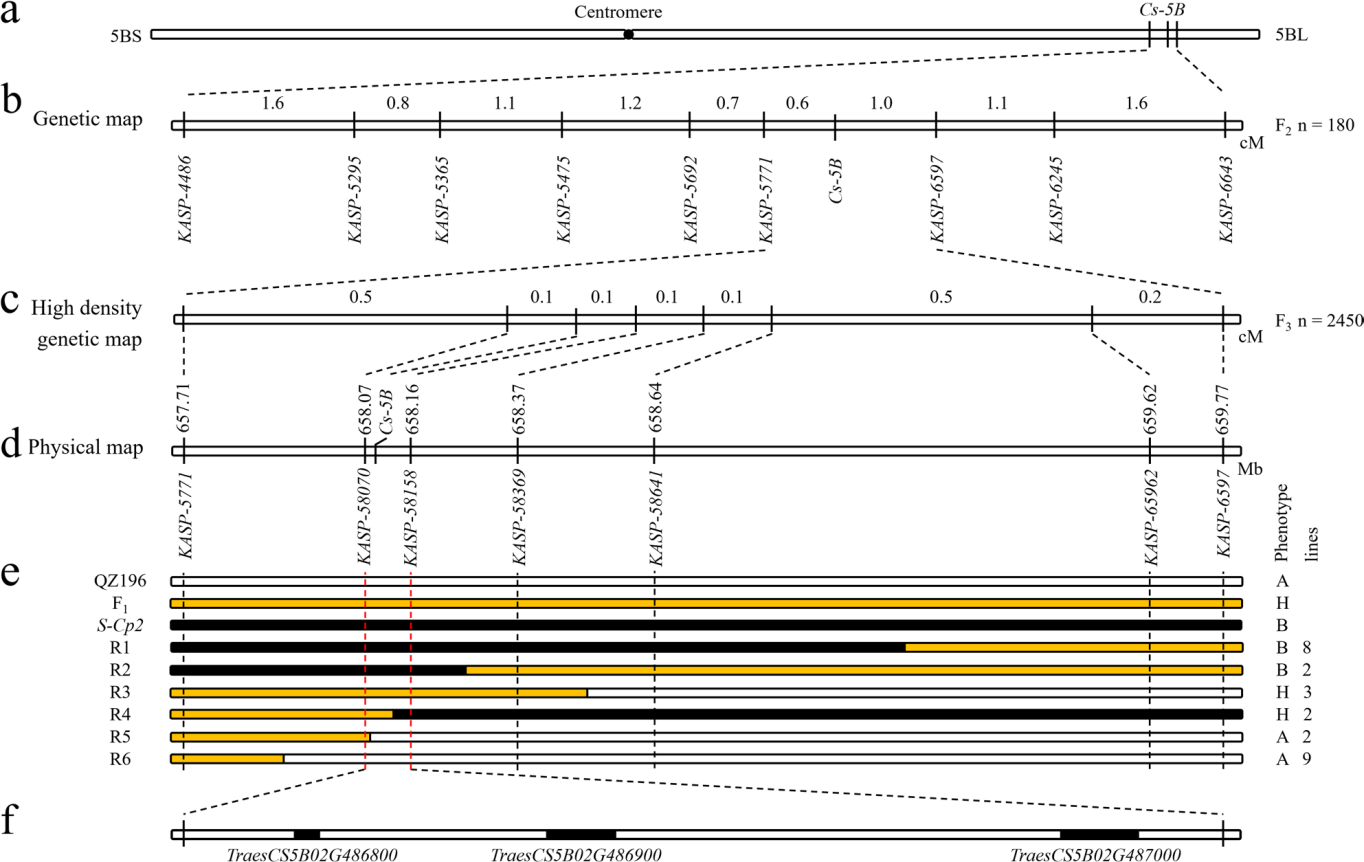
Mapping of the *Cs-5B* locus. **a*** Cs-5B* is located on chromosome 5BL. **b** Mapping of *Cs-5B* using an F_2_ population (‘QZ196’ × *S-Cp2*) consisting of 180 individuals. **c** Fine-mapping of *Cs-5B* using an F_3_ population (‘QZ196’ × *S-Cp2*) consisting of 2,450 individuals. **d** Physical position of newly developed molecular markers used for the fine-mapping of the *Cs-5B* locus. **e** Identification of recombinants. Black, white, and orange segments represent the homozygous allele of *S-Cp2*, homozygous allele of ‘QZ196’, and heterozygous allele, respectively. A, H, and B represent the normal spike phenotype, heterozygous compact spike phenotype, and homozygous compact spike phenotype, respectively. R1–R6 indicate the 6 types of recombinants. There were 8, 2, 3, 2, 2 and 9 lines for R1, R2, R3, R4, R5 and R6, respectively. **f** Three high-confidence predicted genes in the target region of the ‘Chinese Spring’ reference genome

The F_2_ population derived from the ‘QZ196’ × *S-Cp2* cross was used to map the causal locus of the compact spike phenotype of *S-Cp2* according to a BSE-seq analysis. After filtering for read-depth quality, 16,900 SNPs were identified between the normal spike pool and compact spike pool, with a higher density of SNPs concentrated in the 613–672 Mb region of wheat chromosome 5BL (Supplementary Fig. S3). To dissect chromosomal localization of the causal locus, ED analysis was performed. SNPs with depth ≤ 5 were filtered out. We raised the allele frequency ED to a power of 4 (ED^4^) to increase the effect of large ED measurements and decrease the effect of low ED measurements. Loess regression was used to fit the data. Plotting of ED^4^ values along the 21 wheat chromosomes revealed a continuous distribution peak on chromosome 5BL, corresponding to a physical region from 654 Mb to 671 Mb (Supplementary Fig. S3), indicating that the causal locus (named *Cs-5B*) was located on 5BL. Using KASP markers (Supplementary Table S2), *Cs-5B* was mapped between markers *KASP-5771* and *KASP-6597*, with a genetic distance of 1.6 cM (Fig. 2a and b).

To more precisely map *Cs-5B*, 40 F_2_ heterozygous plants from the ‘QZ196’ × *S-Cp2* cross were self-pollinated to generate an F_3_ population consisting of 2,450 individuals. Five newly developed KASP markers (Supplementary Table S2) were used for screening recombinants. Consequently, 26 important recombinant lines were identified and classified into 6 categories (R1–R6; Fig. 2e). There were 8, 2, 3, 2, 2 and 9 lines for R1, R2, R3, R4, R5 and R6, respectively. Based on genotype and phenotype data, *Cs-5B* was delimited to an 87 kb interval between markers *KASP-58070* and *KASP-58158* (Fig. 2c and e). This region included three high-confidence predicted genes (*TraesCS5B02G486800*, *TraesCS5B02G486900*, and *TraesCS5B02G487000*; Fig. 2f).

### *5Bq* is the gene underlying the *Cs-5B* locus

**Fig. 3:**
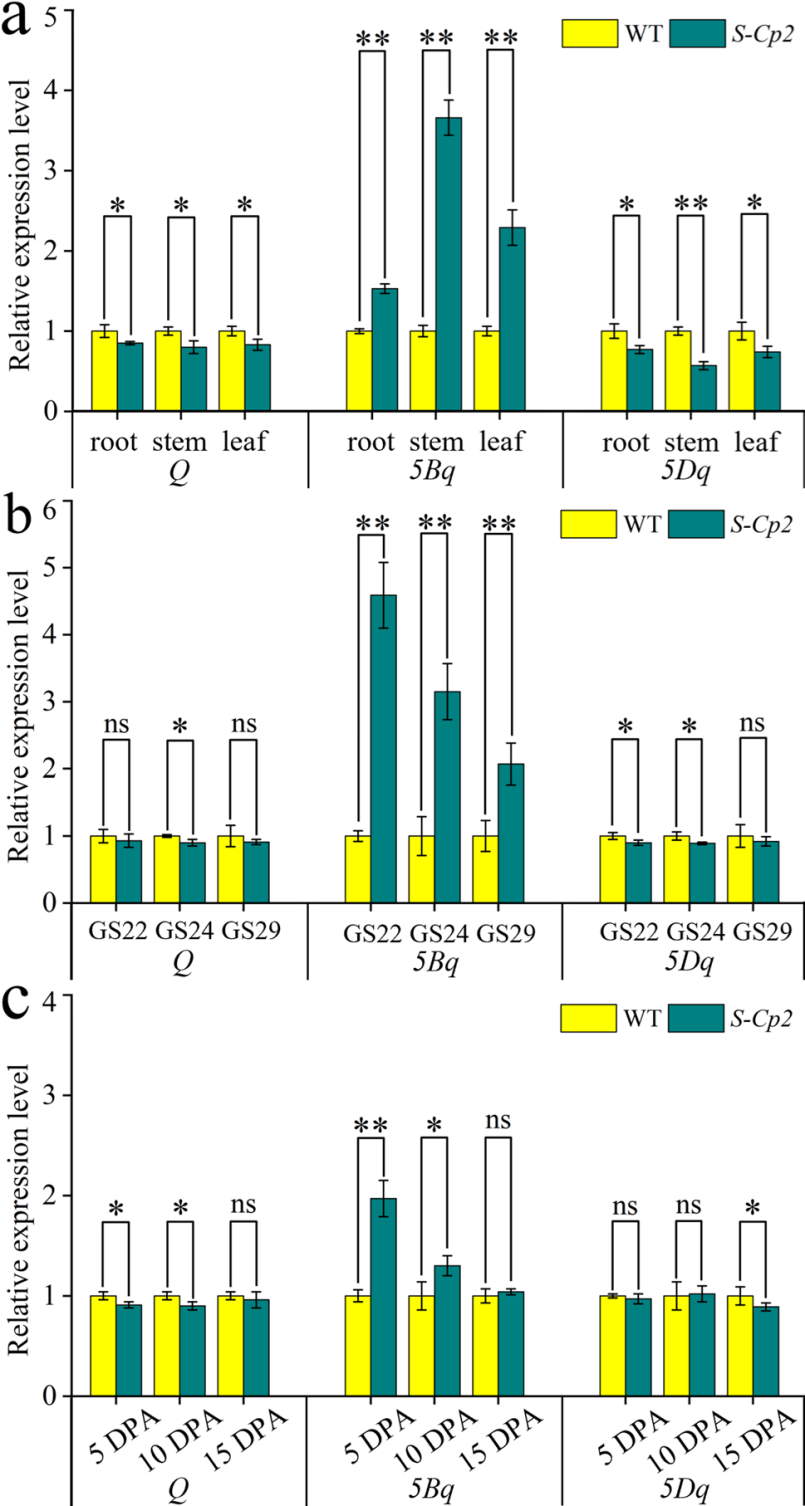
Comparison of *Q*, *5Bq*, and *5Dq* expression levels in the roots, stems, leaves (**a**; GS20), developing spikes (**b**; GS22, GS24, and GS29), and young grains (**c**; 5, 10, and 15 DPA) of *S-Cp2* and WT plants. **, *P* < 0.01; *, *P* < 0.05. Expression levels were determined by qRT-PCR. DPA, days post-anthesis

Analyses of the three high-confidence predicted genes indicated that the *TraesCS5B02G486800* and *TraesCS5B02G487000* sequences in ‘Shumai482’ (i.e., WT) were the same as the corresponding sequences in *S-Cp2*. Therefore, *TraesCS5B02G486900* (*5Bq*), which is homologous to the *Q* gene on 5AL, was hypothesized to be the candidate gene for *Cs-5B*. To test this hypothesis, *5Bq* coding and genomic sequences were cloned from *S-Cp2* and ‘Shumai482’. A missense mutation (G-to-A) was called in the microRNA172-binding site of the *5Bq* coding sequence in *S-Cp2* (Supplementary Figs. S4–S6), resulting in an amino acid change from alanine to threonine. This mutant *5Bq* allele in *S-Cp2* was named *5BQ*^*4*^ (GenBank No. PP584578) because the missense mutation in its microRNA172-binding site was the same as that in the reported *Q*^*c4*^ allele of the *Q* gene (Xu et al., 2018). A qRT-PCR analysis of the roots, stems, leaves, spikes, and grains (Fig. 3) indicated that *5BQ*^*4*^ transcript levels in *S-Cp2* were significantly higher than *5Bq* transcript levels in WT. In addition, *Q* and *5Dq* transcript levels tended to be lower in *S-Cp2* than in WT (Fig. 3). 

**Fig. 4:**
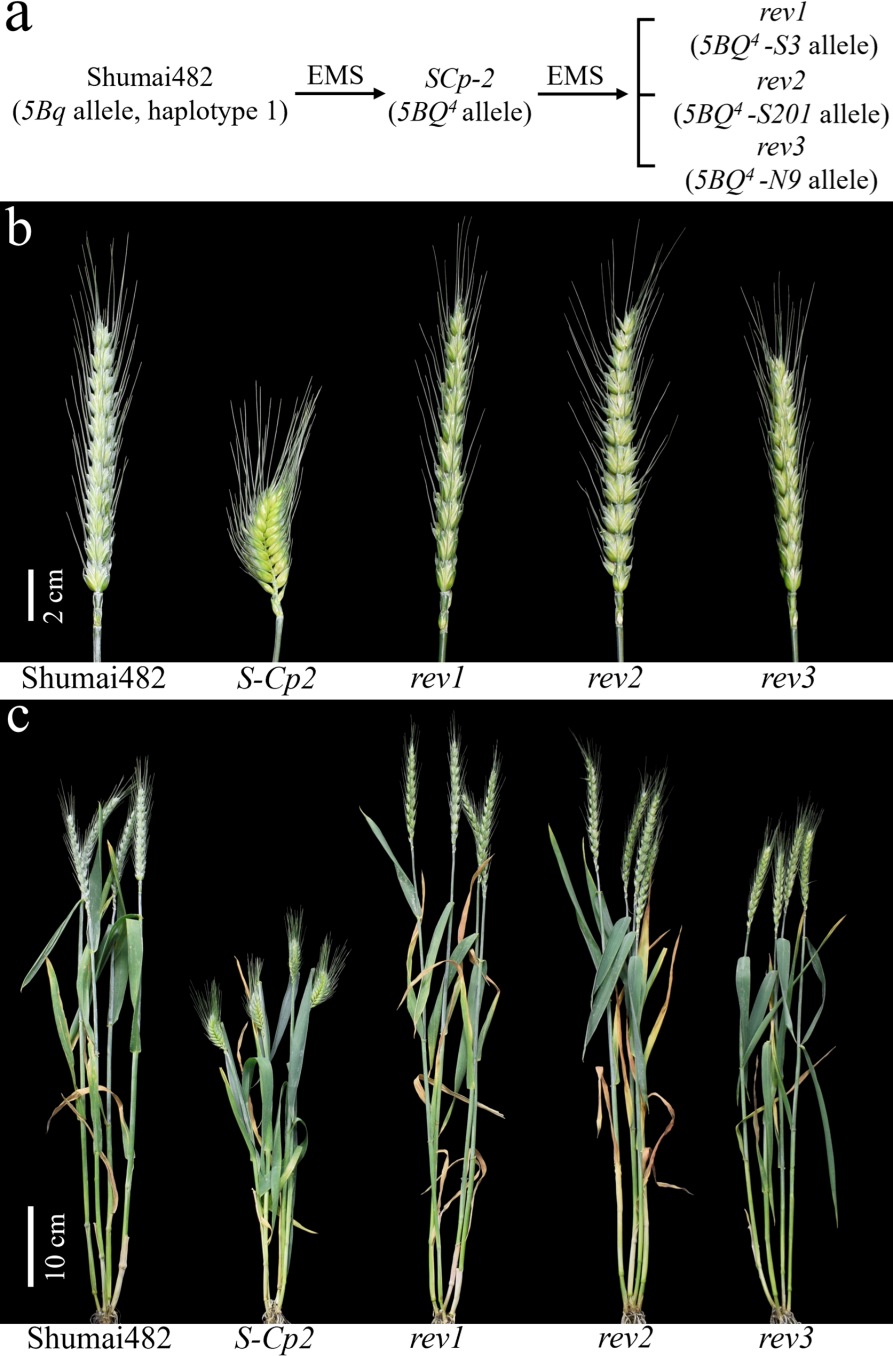
Schematic of the generation of *S-Cp2* and three revertants (*rev1*, *rev2*, and *rev3*) from ‘Shumai482’ (**a**) and comparison of spike morphology (**b**; scale bar, 2 cm) and plant architecture (**c**; scale bar, 10 cm) of ‘Shumai482’, *S-Cp2* and three revertants at GS60

To further assess whether *5Bq* is the gene responsible for the compact spike phenotype, *S-Cp2* seeds were treated with EMS to isolate revertants (Fig. 4a). Consequently, three revertants (*rev1*, *rev2*, and *rev3*) with a normal spike were obtained, which were backcrossed twice with ‘Shumai482’ (Fig. 4). The mutant *5Bq* alleles in *rev1* (nonsense mutation revealed by the comparison with *5BQ*^*4*^ allele), *rev2* (nonsense mutation), and *rev3* (missense mutation) were designated as *5BQ*^*4*^*-S3* (GenBank No. PP584579), 5*BQ*^*4*^*-S201* (PP584581), and *5BQ*^*4*^*-N9* (PP584580), respectively (Supplementary Figs. S4–S6). Compared with *S-Cp2*, the three revertants had a normal spike and were taller (Fig. 4b and c; Supplementary Table S4).

### *5Bq* alleles act as a nucleus-localized factor

**Fig. 5:**
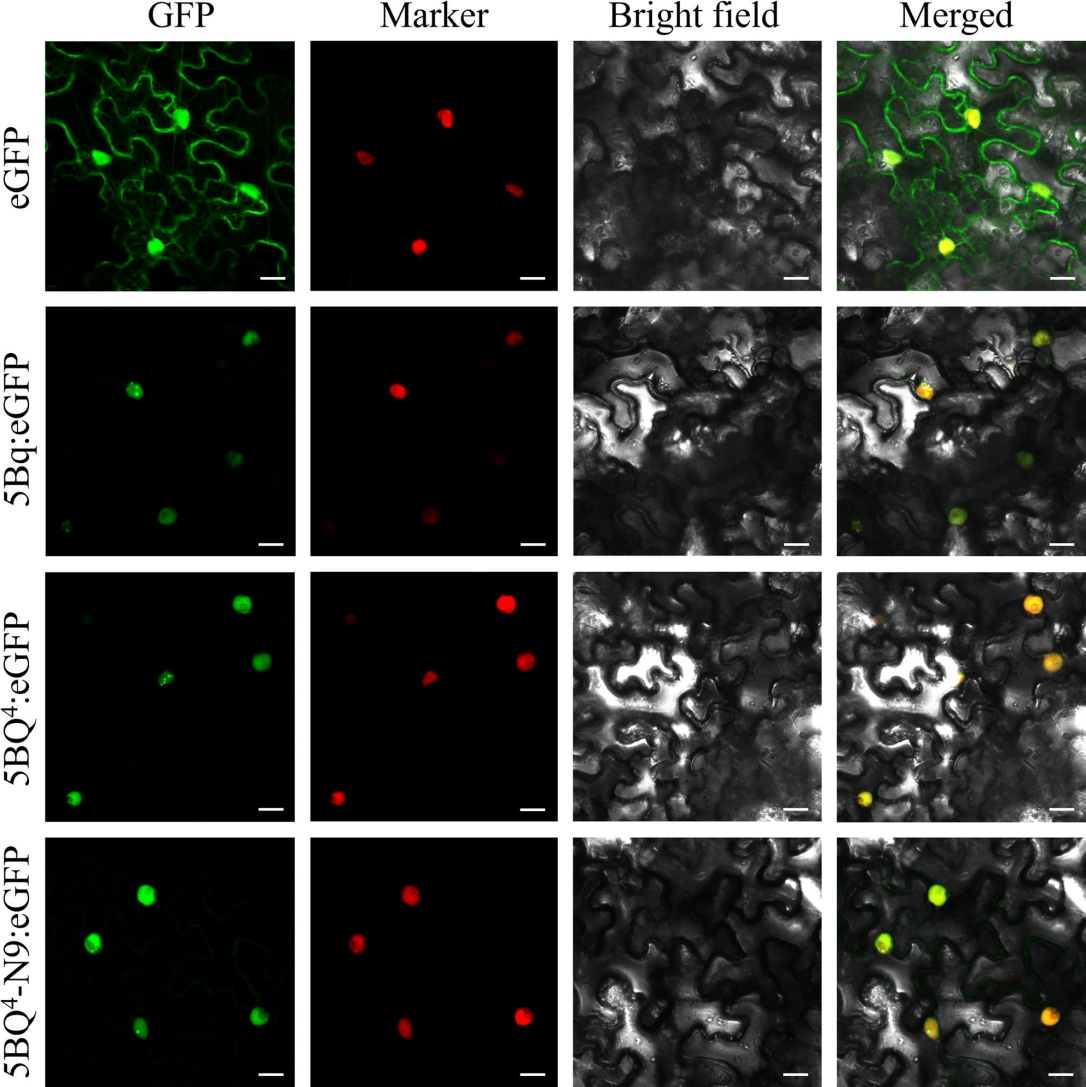
Subcellular localization of 5Bq, 5BQ^4^ and 5BQ^4^-N9 proteins in *N. benthamiana* leaves. eGFP, 5Bq: eGFP, 5BQ^4^:eGFP, and 5BQ^4^-N9:eGFP represent pCAMBIA2300-eGFP, pCAMBIA2300-5Bq-eGFP, pCAMBIA2300-5BQ^4^-eGFP and pCAMBIA2300-5BQ^4^-N9-eGFP vectors, respectively. GFP, images of the green fluorescence channel. Marker, images of nucleus marker protein fluorescence channel. Bright field, images of white light bright field. Merged, merged images of GFP, Marker, and Bright field. Scale bar, 20 μm

To determine their subcellular localization, the fused 5Bq-eGFP, 5BQ4-eGFP, and 5BQ4-N9-eGFP proteins were expressed in *N. benthamiana* leaves, respectively. Microscopic examinations indicated that 5Bq, 5BQ4 and 5BQ4-N9 were specifically distributed in the nucleus of the resulting tobacco cells, whereas the contrasted eGFP was distributed throughout the cells (Fig. 5). 

### Haplotype analysis

**Fig. 6:**
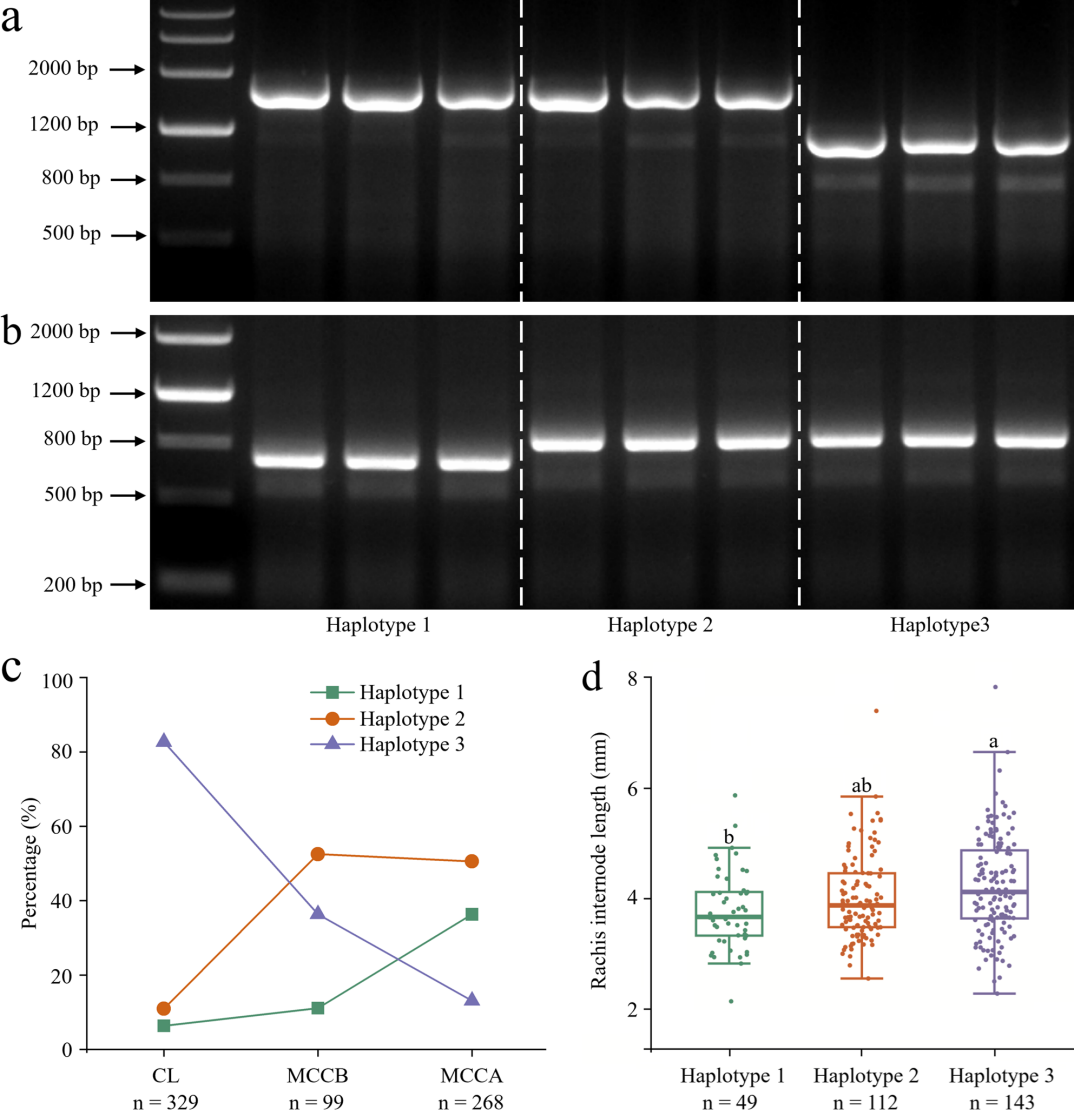
Haplotype analysis for *5Bq* gene. Utility of the markers *5BqDNA-1* (**a**) and *5BqDNA-3* (**b**) for distinguishing *5Bq* haplotype 1 (GenBank No. PP584577), haplotype 2 (PP584582), and haplotype 3 (PP584583). Genomic DNA was used as the PCR template. PCR products were separated on a 1.5% agarose gel. Distribution of the three haplotypes of *5Bq* (**c**) and comparison of their effects on rachis internode length (**d**) in common wheat, including Chinese landraces (CL), modern Chinese cultivars released before the year of 2000 (MCCB), and modern Chinese cultivars released after 2000 (MCCA). Lowercase letters above the column indicate significance at *P* < 0.05. ‘n’ indicates the number of wheat materials used for analysis

To clarify haplotype variations, *5Bq* coding and genomic sequences in 30 common wheat accessions were cloned and analyzed. Three haplotypes (haplotypes 1 [functional], 2 [pseudogene], and 3 [pseudogene]) were identified (Supplementary Figs. S7‒S9). After the coding sequences were aligned (Supplementary Fig. S8), frameshift mutations were detected within the first AP2 domain in haplotypes 2 and 3, which prevented the translation of full-length 5Bq proteins (Supplementary Figs. S8 and S9). Two markers (*5BqDNA-1* and *5BqDNA-3*) were developed on the basis of indels in the genomic sequences of the three haplotypes (Supplementary Fig. S7). *5BqDNA-1* and *5BqDNA-3* were specific for haplotypes 3 and 1, respectively. The haplotypes could be distinguished via the combined use of *5BqDNA-1* and *5BqDNA-3* (Fig. 6a and b). We used the genomic DNA of 696 common wheat materials to perform a haplotype analysis for *5Bq* (Supplementary Table S1). The proportion of functional haplotype 1 increased from 6.38% in CL to 11.11% in MCCB and then to 36.57% in MCCA (Fig. 6c), indicating that it was gradually accumulated in Chinese wheat cultivars. Notably, haplotype 1 is associated with a smaller rachis internode length than haplotypes 2 (with no significance) and 3 (with significance at *P* < 0.05) (Fig. 6d), even though we can not fully exclude the effects of other genetic loci. The deduced peptide of haplotype 2 is shorter and longer than those of haplotypes 1 and 3 (Supplementary Fig. S9), respectively. Therefore, we speculate that haplotype 2 may retain a weaker effect than haplotype 1 and a stronger effect than haplotype 3 on rachis internode length.

## Discussion

### *S-Cp2* mutant phenotype is due to an overexpressed *5Bq* allele

Common wheat is hexaploid (AABBDD; 2n = 6x = 42) that contains three homologous genomes. Overexpression of *Q* on chromosome 5AL leads to increases in TKW, but a decrease in plant height and rachis internode length [41, 44, 59]. In this study, compared with the WT control, *S-Cp2* mutant plants had a more compact spike and larger grains, but they were shorter as expected (Fig. 1; Supplementary Table S3). According to a correlation analysis, rachis internode length was positively correlated with rachis length and plant height, but negatively correlated with TKW (Supplementary Table S5).

MicroRNAs regulate gene expression mainly by promoting translational repression and mRNA degradation [44, 60–62]. Base pairing between microRNAs and their target sites is crucial for mRNA cleavage [63]. Point mutations in the microRNA172-binding site of *Q/5Dq* lead to increase *Q/5Dq* transcripts by reducing microRNA172 cleavage efficiency. The resulting increase in *Q/5Dq* expression affects multiple traits, including rachis internode length, plant height, and TKW [32, 41, 45]. Similarly, point mutations within the microRNA172-binding site in wheat orthologs of barley *Cly1* decrease the likelihood of microRNA-guided cleavage and suppress lodicule development in hexaploid wheat [48, 64, 65].

*S-Cp2* is a common wheat mutant with a compact spike. The gene controlling the compact spike phenotype was mapped to an 87 kb interval on chromosome 5BL, where *5Bq* (homologous to *Q* on 5AL and *5Dq* on 5DL) was located. One missense mutation was called within the microRNA172-binding site of *5Bq* in *S-Cp2* (i.e., *5BQ*^*4*^ allele). As expected, *5BQ*^*4*^ transcription in *S-Cp2* exceeded *5Bq* transcription in WT (Fig. 3). Based on the observations in the *Q/5Dq* gene, we infer that the missense mutation in microRNA172-binding site of *5Bq* impairs microRNA172-mediated cleavage efficiency, which consequently leads to the elevated expression of *5Bq* gene [32, 41, 45]. Furthermore, the revertants with non-functional *5Bq* alleles (*5BQ*^*4*^*-S3* and *5BQ*^*4*^*-S201*; Figs. 4; Supplementary Figs. S4–S6) had normal plant morphological characteristics. These observations suggest *5BQ*^*4*^ is an overexpressed *5Bq* allele that is responsible for the compact spike phenotype of *S-Cp2*.

### *5Bq* is a potentially valuable target for breeders

Zhang et al. [46] identified *5Bq* as a pseudogene in *T. aestivum* cv. Chinese Spring (haplotype 3) and in the tetraploid *Triticum turgidum* (haplotype 2) [46]. In 2011, the genome sequencing data of wheat accessions were still unavailable, which possibly led to the failure in the identification of functional haplotype 1. In the current study, we reported the functional haplotype 1 for *5Bq* (Supplementary Figs. S7–S9; Supplementary Table S1). In addition, the difference within the AP2 domain of the three haplotypes was verified by an analysis of published wheat cultivar reference sequences [66, 67]. The proportion of haplotype 1 increased from 6.38% in CL to 36.57% in MCCB, suggestive of the long-term existence of haplotype 1 in cultivated wheat as well as the gradual accumulation of this haplotype during the wheat breeding process.

Compared with the plants with non-functional alleles derived from *5BQ*^*4*^ (*5BQ*^*4*^*-S3* and *5BQ*^*4*^*-S201*; Figs. 4; Supplementary Figs. S4–S6), the plants with functional alleles (*5BQ*^*4*^ and *5BQ*^*4*^*-N9*) were shorter (Fig. 4; Supplementary Table S4), suggesting that functional *5Bq* may be useful for decreasing the risk of lodging. Therefore, we speculate that haplotype 1 was selected by breeders to decrease plant height. These results reflect the potential utility of *5Bq* for wheat breeding. However, additional favorable *5Bq* allele(s) will need to be identified on the basis of *5BQ*^*4*^.

### Generating favorable *5Bq* alleles for breeding

Similar to the reported *Q*^*c1*^ allele on 5AL [41], *5BQ*^*4*^ on 5BL was associated with the compact spike phenotype (Fig. 1), which is an unfavorable trait in most wheat-growing areas. Introducing amino acid changes in the AP2 domain of the protein encoded by *Q* on 5AL can lead to increases in rachis internode length [32, 41, 42]. Hence, EMS was used to generate the favorable *Q*^*c1*^*-N8* and *Q*^*c1*^*-Q1* alleles from *Q*^*c1*^ [42, 68]. A comparison with *Q*^*c1*^ revealed *Q*^*c1*^*-N8* contains a missense mutation in its second AP2-encoding domain; this mutation results in simultaneous increases in the grain protein content and grain yield [42]. A comparison with *Q*^*c1*^ revealed *Q*^*c1*^*-Q1* contains a point mutation in the splice site of the second intron; this mutation leads to an increase in yield with no penalty on grain quality [68]. A clarification of underlying mechanisms through DNA affinity purification sequencing and transcriptome sequencing analyses, indicated that genes related to photosynthesis or carbon and nitrogen metabolism were enriched and positively regulated by Q, which provides molecular evidence of the positive regulatory effects of Q on wheat yield and quality [69]. Similarly, we obtained *5BQ*^*4*^*-N9* by introducing a point mutation in the first AP2-encoding domain of *5BQ*^*4*^ (Supplementary Figs. S4–S6). Wheat plants carrying *5BQ*^*4*^*-N9* showed lower plant height and greater grain number per spike than plants with non-functional *5Bq* alleles (*5BQ*^*4*^*-S3* and *5BQ*^*4*^*-S201*; Fig. 4; Supplementary Table S4), indicating the value of generating favorable *5Bq* alleles in the following experiment by chemical mutagenesis and CRISPR technology [70].

## Conclusion

In summary, *S-Cp2* is a common wheat mutant with a compact spike. The result of a genetic analysis indicated that the compact spike phenotype was due to a single dominant locus (*Cs-5B*) on chromosome 5BL. More specifically, *Cs-5B* was precisely mapped to an 87 kb interval containing three high-confidence predicted genes, including 5*Bq* (*TraesCS5B02G486900*). One missense mutation was found within the microRNA172-binding site of 5*Bq* (i.e., *5BQ*^*4*^ allele), which is responsible for the mutant phenotype of *S-Cp2*. As expected, the 5*BQ*^*4*^ transcript level was significantly higher than that of the 5*Bq* allele. According to a haplotype analysis, the functional haplotype 1 for *5Bq* was gradually accumulated in modern Chinese wheat cultivars. Considered together, the research findings highlight the potential value of 5*Bq* in wheat breeding programs.

## Supplementary Information

Supplementary Material 1: Fig. S1. Schematic of the generation of the *S-Cp2* mutant with the genetic background of common wheat cultivar ‘Shumai482’ (a) and the construction of segregating populations (b).

Supplementary Material 2: Frequency (percentage) distribution histogram and Gaussian curve (red line) for the rachis internode length of plants in the ‘Shumai482’ × *S-Cp2* BC_1_F_2_ (a), ‘QZ196’ × *S-Cp2* F_2_ (b), and ‘Mianmai112’ × *S-Cp2* F_2_ (c) populations. Yellow and blue indicate the percentages of plants with normal and compact spikes, respectively. The horizontal axis shows the rachis internode length (mm).

Supplementary Material 3: Fig. S3. Mapping of the *Cs-5B* locus using SNPs on the basis of the bulked segregant analysis coupled with exome capture sequencing of two DNA pools derived from plants with a compact spike and plants with a normal spike. (a) SNP density index of the normal spike DNA pool and compact spike DNA pool. Each vertical line represents the number of SNPs in a 1 Mb segment. SNP density decreases from high (red) to low (green). (b) Euclidean distance (ED) analysis. The allele frequency ED to a power of 4 (ED^4^) to increase the effect of large ED measurements and decrease the effect of low ED measurements. The upper and lower figures were drawn on the basis of the fitted and original data, respectively. Dots represent the ED^4^ value of SNPs. Loess fit curves for ED^4^ revealed a continuous distribution peak on chromosome 5BL, suggesting that causal locus of the compact spike phenotype of *S-Cp2* was located in 5BL.

Supplementary Material 4: Fig. S4. Alignment of the deduced amino acid sequences encoded by *5Bq* (GenBank No. PP584577), *5BQ*^*4*^ (PP584578), *5BQ*^*4*^*-S3* (PP584579), *5BQ*^*4*^*-S201* (PP584581), and *5BQ*^*4*^*-N9* (PP584580). Seven previously described conserved domains (motif 1, motif 2, nuclear localization signal, AP2 domain R1, AP2 domain R2, motif 3, and AASSGF box) are presented. Asterisks indicate the stop codon. Red arrows indicate the amino acid substitutions caused by missense mutations.

Supplementary Material 5: Fig. S5. Alignment of the coding sequences of *5Bq* (GenBank No. PP584577), *5BQ*^*4*^(PP584578), *5BQ*^*4*^*-S3* (PP584579), *5BQ*^*4*^*-S201* (PP584581), and *5BQ*^*4*^*-N9* (PP584580) alleles. The microRNA172-binding site and sequences of seven conserved domains (motif 1, motif 2, nuclear localization signal, AP2 domain R1, AP2 domain R2, motif 3, and AASSGF box) are annotated. Red arrows indicate point mutations.

Supplementary Material 6: Fig. S6. Alignment of the genomic sequences of *5Bq* (GenBank No. PP584577), *5BQ*^*4*^(PP584578), *5BQ*^*4*^*-S3* (PP584579), *5BQ*^*4*^*-S201* (PP584581), and *5BQ*^*4*^*-N9* (PP584580). The microRNA172-binding site, 10 exons, and nine introns are annotated. Red arrows indicate point mutations.

Supplementary Material 7: Fig. S7. Differences in the genomic sequences of *5Bq* haplotype 1 (GenBank No. PP584577), haplotype 2 (PP584582), and haplotype 3 (PP584583). Ten exons and nine introns are annotated.

Supplementary Material 8: Fig. S8. Differences in the coding sequences of *5Bq* haplotype 1 (GenBank No. PP584577), haplotype 2 (PP584582), and haplotype 3 (PP584583). The sequences of seven conserved domains (motif 1, motif 2, nuclear localization signal, AP2 domain R1, AP2 domain R2, motif 3, and AASSGF box) are annotated.

Supplementary Material 9: Fig. S9. Alignment of the deduced amino acid sequences encoded by *5Bq* haplotype 1 (GenBank No. PP584577), haplotype 2 (PP584582), and haplotype 3 (PP584583). Seven previously described conserved domains (motif 1, motif 2, nuclear localization signal, AP2 domain R1, AP2 domain R2, motif 3, and AASSGF box) are annotated. Asterisks indicate the stop codon.

Supplementary Material 10.

## Data Availability

BSE-seq data generated during the current study are available in the Genome Sequence Archive at the Beijing Institute of Genomics (BIG) Data Center (CRA037883; https://ngdc.cncb.ac.cn/gsa).

## Abbreviations

AP2: Apetala2
BSE-seq: Bulked segregant analysis coupled with exome capture sequencing
CL: Chinese landraces
DPA: Days post anthesis
ED: Euclidean distance
eGFP: Enhanced green fluorescent protein
EMS: Ethyl methanesulfonate
KASP: Kompetitive allele-specific PCR
MCCA: Modern chinese cultivars released after 2000
MCCB: Modern chinese cultivars released before 2000
SNP: Single nucleotide polymorphism
TKW: Thousand kernel weight
