# Supplementary figures and images for: Functional 5Bq allele is responsible for the compact spike phenotype in common wheat (Triticum aestivum L.)
Source: BMC Plant Biol. 2026 Mar 17;26:746. doi: 10.1186/s12870-026-08583-x (PMC13107888; doi:10.1186/s12870-026-08583-x)

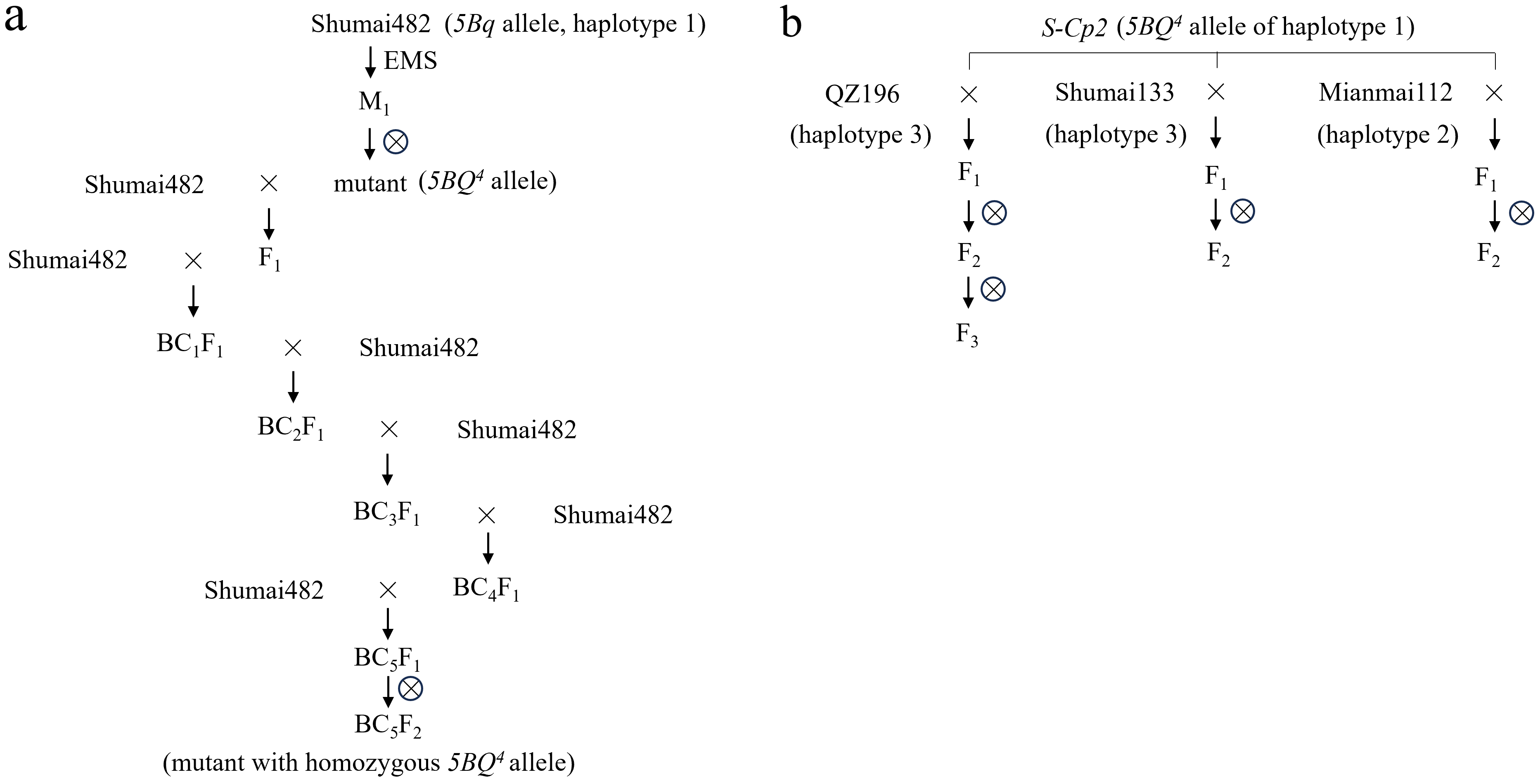

Supplement: Supplementary file 1 — Supplementary Material 1: Fig. S1. Schematic of the generation of the S-Cp2 mutant with the genetic background of common wheat cultivar ‘Shumai482’ (a) and the construction of segregating populations (b). [file 12870_2026_8583_MOESM1_ESM.tif]

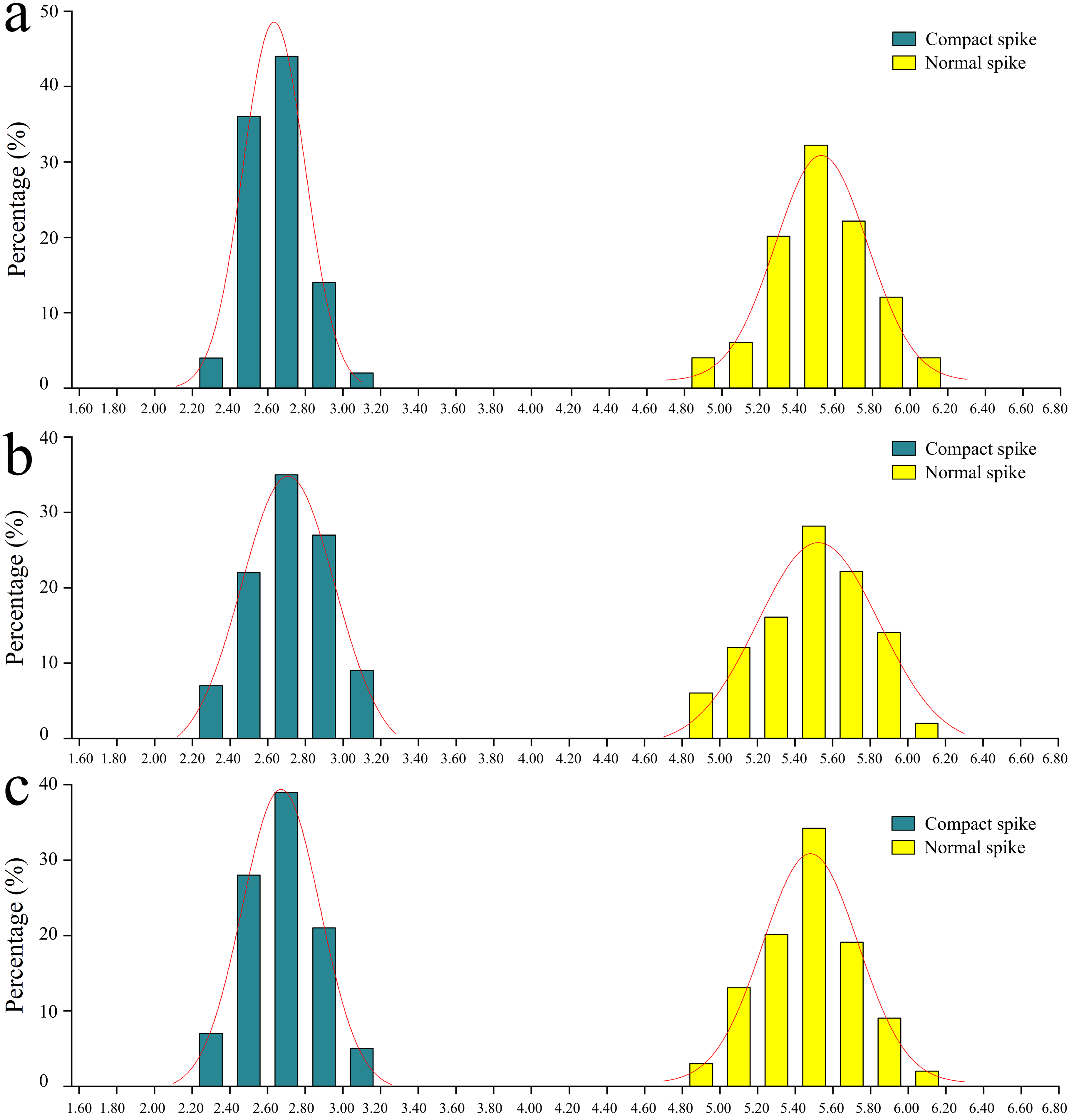

Supplement: Supplementary file 2 — Supplementary Material 2: Frequency (percentage) distribution histogram and Gaussian curve (red line) for the rachis internode length of plants in the ‘Shumai482’ × S-Cp2 BC1F2 (a), ‘QZ196’ × S-Cp2 F2 (b), and ‘Mianmai112’ × S-Cp2 F2 (c) populations. Yellow and blue indicate the percentages of plants with normal and compact spikes, respectively. The horizontal axis shows the rachis internode length (mm). [file 12870_2026_8583_MOESM2_ESM.tif]

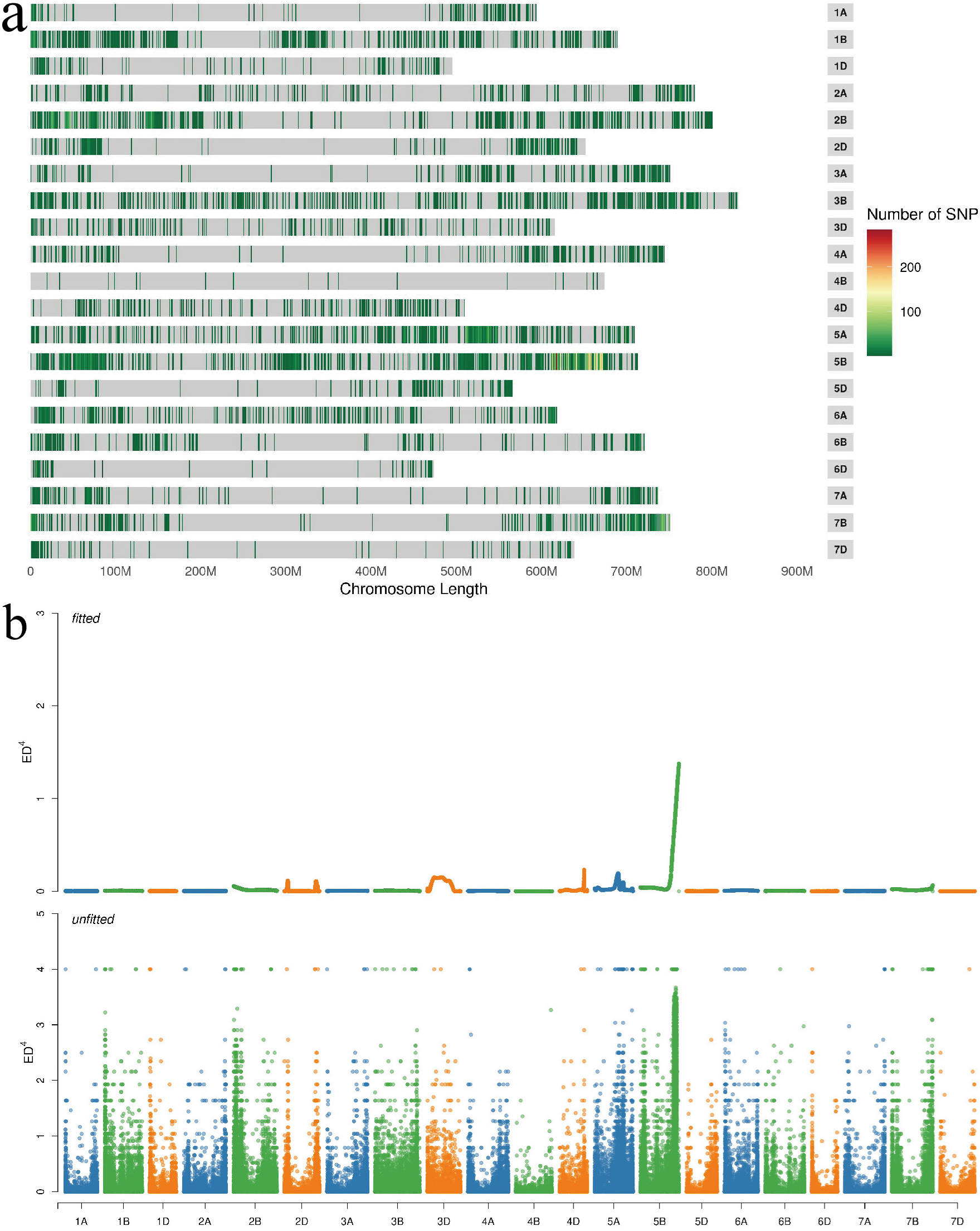

Supplement: Supplementary file 3 — Supplementary Material 3: Fig. S3. Mapping of the Cs-5B locus using SNPs on the basis of the bulked segregant analysis coupled with exome capture sequencing of two DNA pools derived from plants with a compact spike and plants with a normal spike. (a) SNP density index of the normal spike DNA pool and compact spike DNA pool. Each vertical line represents the number of SNPs in a 1 Mb segment. SNP density decreases from high (red) to low (green). (b) Euclidean distance (ED) analysis. The allele frequency ED to a power of 4 (ED4) to increase the effect of large ED measurements and decrease the effect of low ED measurements. The upper and lower figures were drawn on the basis of the fitted and original data, respectively. Dots represent the ED4 value of SNPs. Loess fit curves for ED4 revealed a continuous distribution peak on chromosome 5BL, suggesting that causal locus of the compact spike phenotype of S-Cp2 was located in 5BL. [file 12870_2026_8583_MOESM3_ESM.tif]

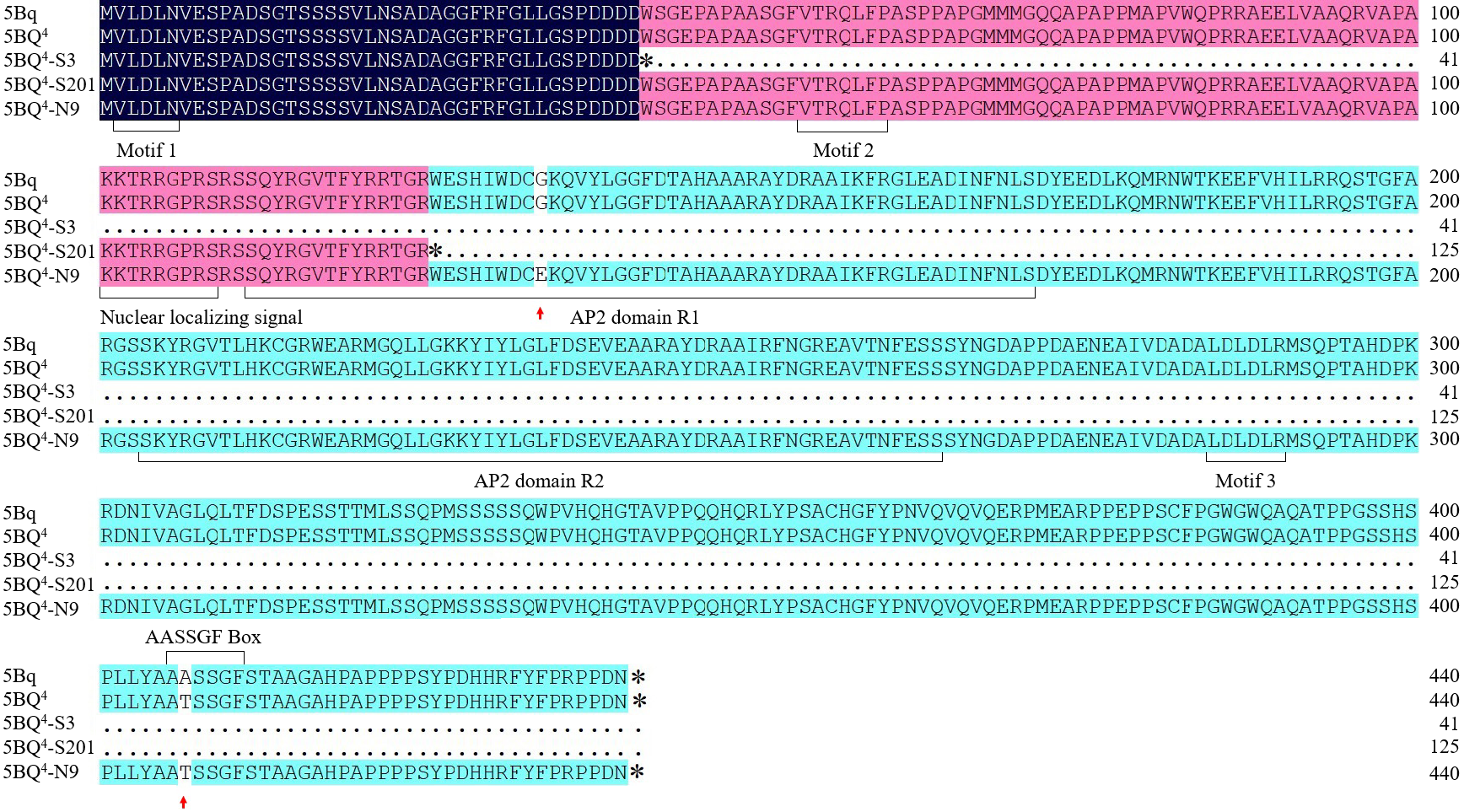

Supplement: Supplementary file 4 — Supplementary Material 4: Fig. S4. Alignment of the deduced amino acid sequences encoded by 5Bq (GenBank No. PP584577), 5BQ4 (PP584578), 5BQ4-S3 (PP584579), 5BQ4-S201 (PP584581), and 5BQ4-N9 (PP584580). Seven previously described conserved domains (motif 1, motif 2, nuclear localization signal, AP2 domain R1, AP2 domain R2, motif 3, and AASSGF box) are presented. Asterisks indicate the stop codon. Red arrows indicate the amino acid substitutions caused by missense mutations. [file 12870_2026_8583_MOESM4_ESM.tif]

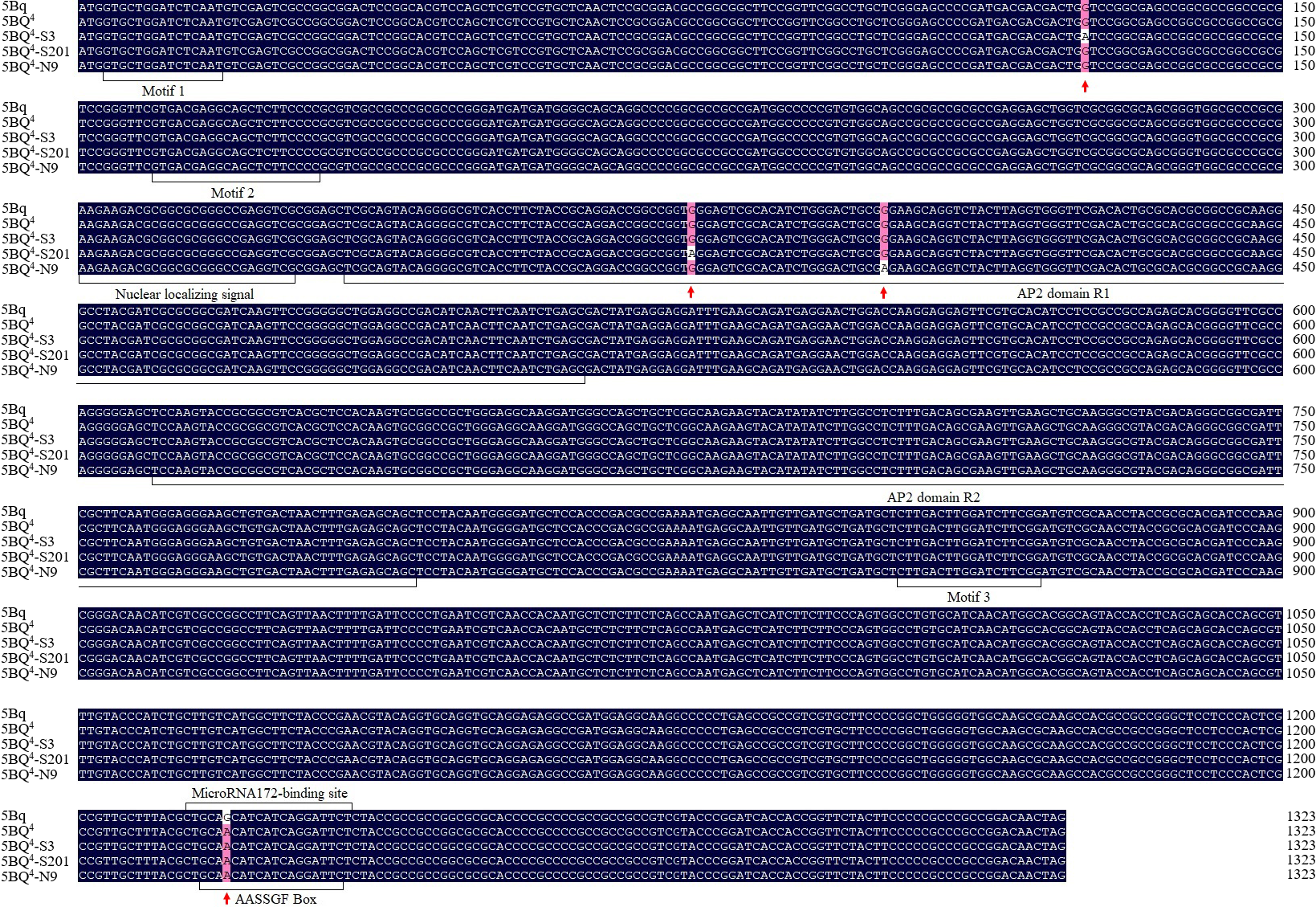

Supplement: Supplementary file 5 — Supplementary Material 5: Fig. S5. Alignment of the coding sequences of 5Bq (GenBank No. PP584577), 5BQ4(PP584578), 5BQ4-S3 (PP584579), 5BQ4-S201 (PP584581), and 5BQ4-N9 (PP584580) alleles. The microRNA172-binding site and sequences of seven conserved domains (motif 1, motif 2, nuclear localization signal, AP2 domain R1, AP2 domain R2, motif 3, and AASSGF box) are annotated. Red arrows indicate point mutations. [file 12870_2026_8583_MOESM5_ESM.tif]

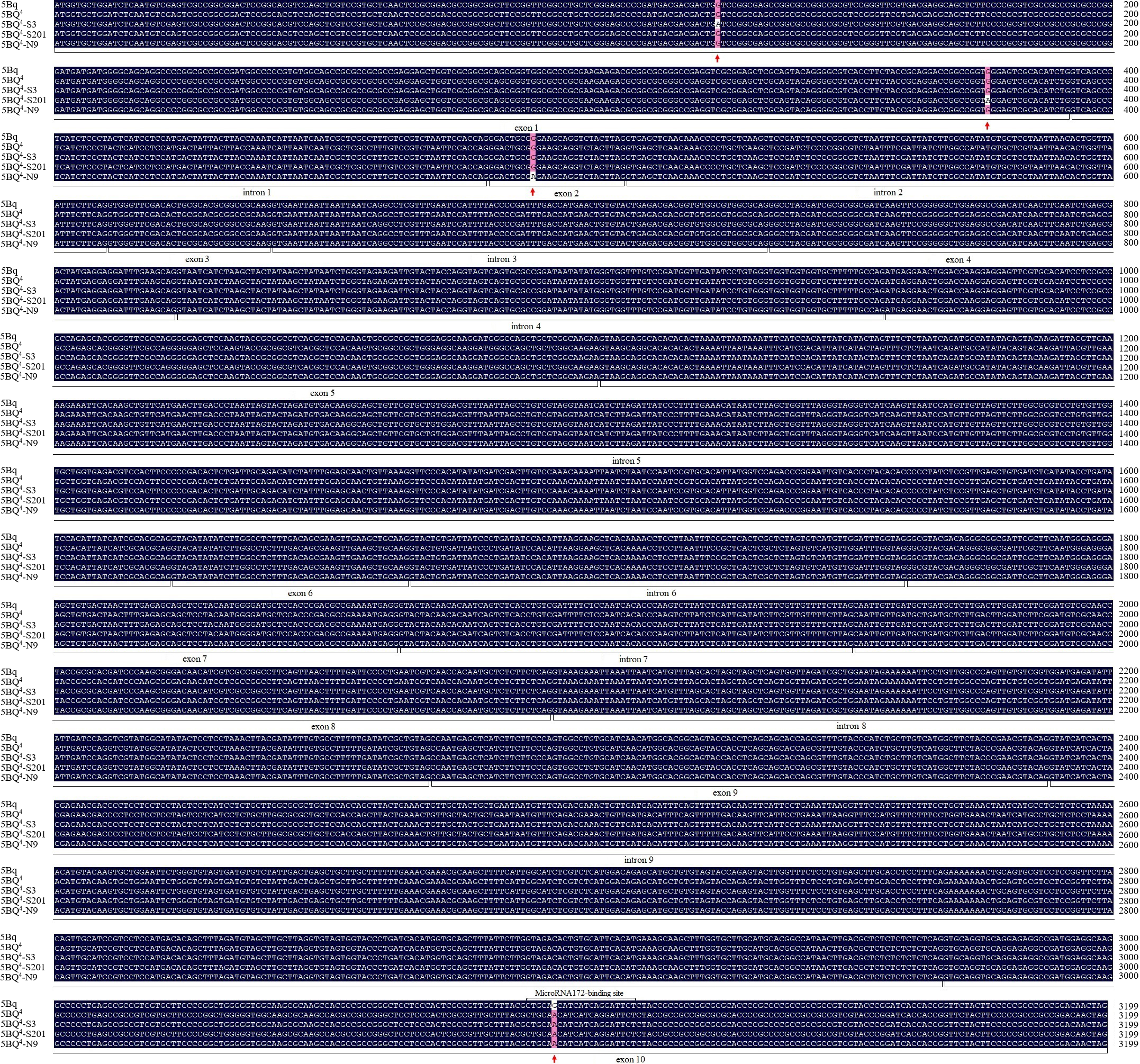

Supplement: Supplementary file 6 — Supplementary Material 6: Fig. S6. Alignment of the genomic sequences of 5Bq (GenBank No. PP584577), 5BQ4(PP584578), 5BQ4-S3 (PP584579), 5BQ4-S201 (PP584581), and 5BQ4-N9 (PP584580). The microRNA172-binding site, 10 exons, and nine introns are annotated. Red arrows indicate point mutations. [file 12870_2026_8583_MOESM6_ESM.tif]

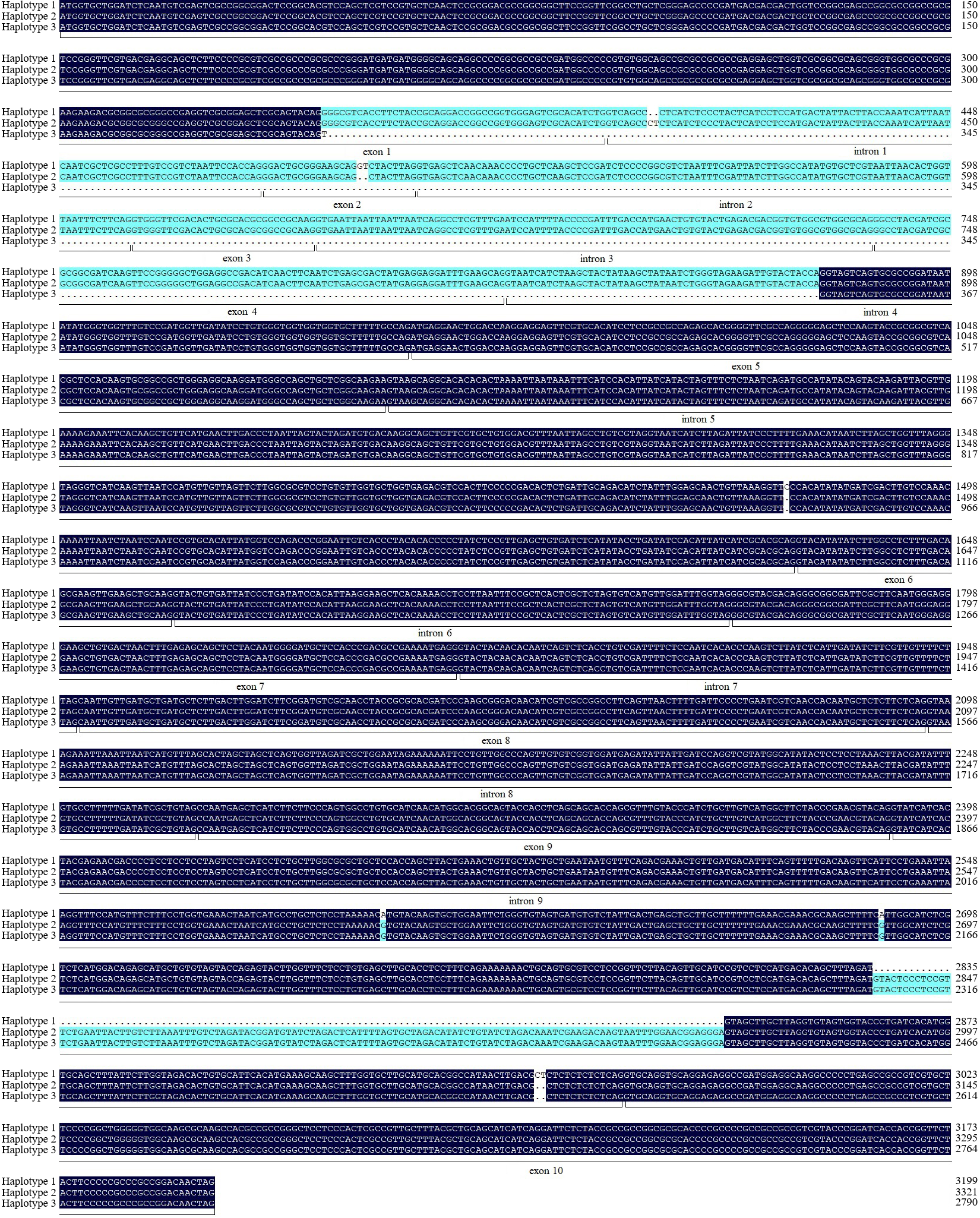

Supplement: Supplementary file 7 — Supplementary Material 7: Fig. S7. Differences in the genomic sequences of 5Bq haplotype 1 (GenBank No. PP584577), haplotype 2 (PP584582), and haplotype 3 (PP584583). Ten exons and nine introns are annotated. [file 12870_2026_8583_MOESM7_ESM.tif]

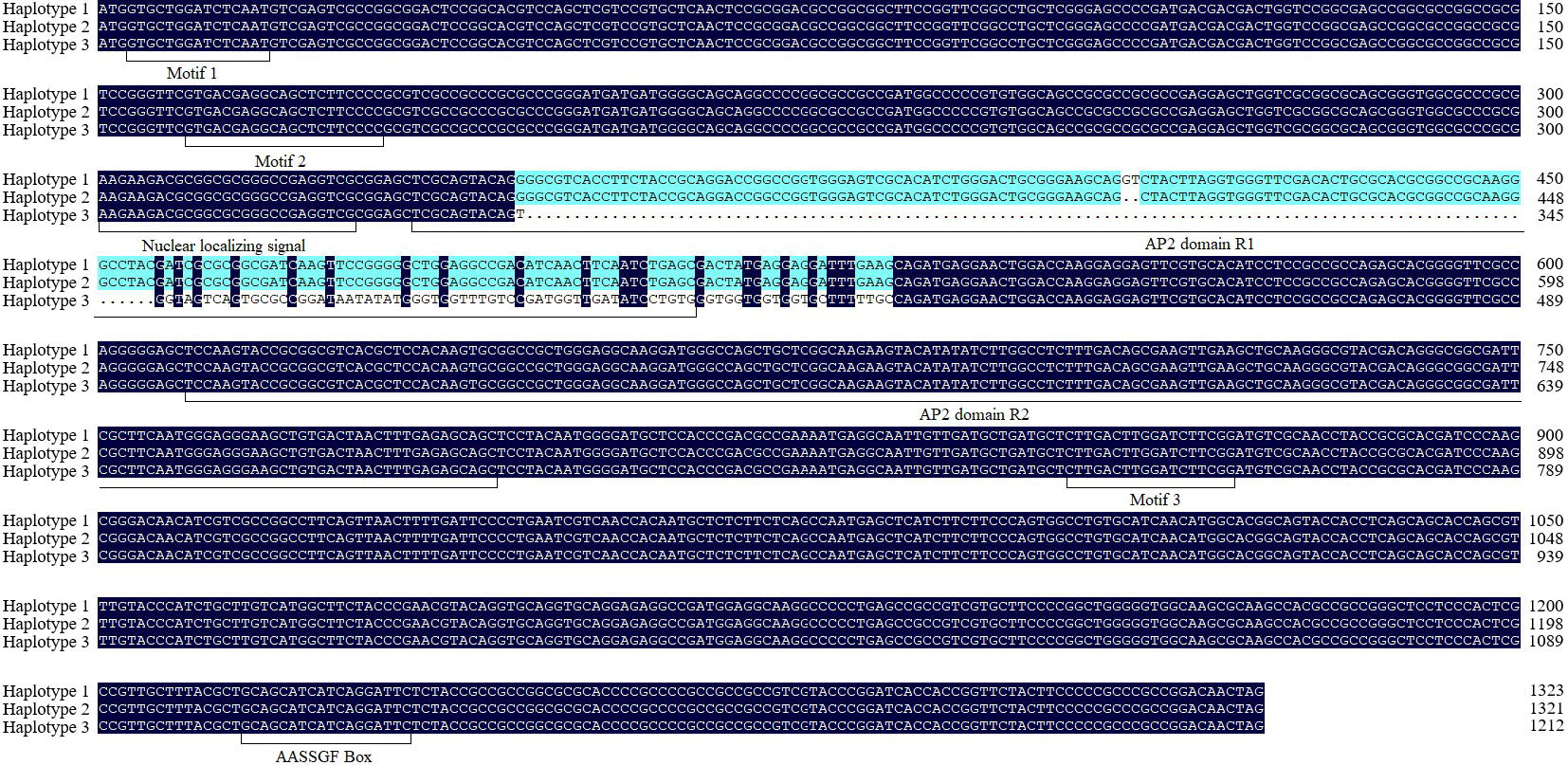

Supplement: Supplementary file 8 — Supplementary Material 8: Fig. S8. Differences in the coding sequences of 5Bq haplotype 1 (GenBank No. PP584577), haplotype 2 (PP584582), and haplotype 3 (PP584583). The sequences of seven conserved domains (motif 1, motif 2, nuclear localization signal, AP2 domain R1, AP2 domain R2, motif 3, and AASSGF box) are annotated. [file 12870_2026_8583_MOESM8_ESM.tif]

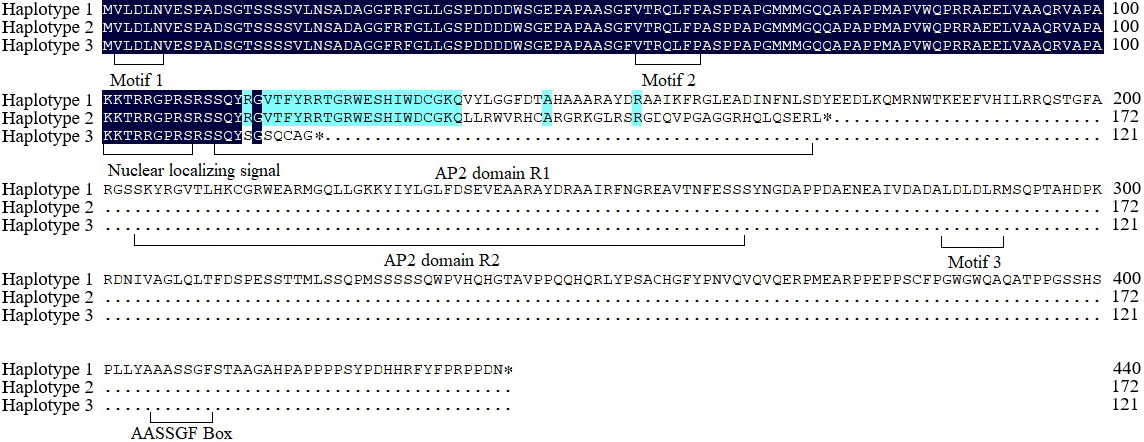

Supplement: Supplementary file 9 — Supplementary Material 9: Fig. S9. Alignment of the deduced amino acid sequences encoded by 5Bq haplotype 1 (GenBank No. PP584577), haplotype 2 (PP584582), and haplotype 3 (PP584583). Seven previously described conserved domains (motif 1, motif 2, nuclear localization signal, AP2 domain R1, AP2 domain R2, motif 3, and AASSGF box) are annotated. Asterisks indicate the stop codon. [file 12870_2026_8583_MOESM9_ESM.tif]
